# Interdisciplinary approach at the primary healthcare level for Bolivian immigrants with Chagas disease in the city of São Paulo

**DOI:** 10.1371/journal.pntd.0005466

**Published:** 2017-03-23

**Authors:** Maria Aparecida Shikanai Yasuda, Camila Gonçalves Sátolo, Noemia Barbosa Carvalho, Magda Maya Atala, Rosario Quiroga Ferrufino, Ruth Moreira Leite, Célia Regina Furucho, Expedito Luna, Rubens Antonio Silva, Marcia Hage, Caroline Medeji Ramos de Oliveira, Felipe Delatorre Busser, Vera Lucia Teixeira de Freitas, Dalva Marli Valerio Wanderley, Luzia Martinelli, Sonia Regina Almeida, Pedro Albajar Viñas, Nivaldo Carneiro

**Affiliations:** 1 Department of Infectious and Parasitic Diseases, Faculdade de Medicina of the University of São Paulo, São Paulo, Brazil; 2 Laboratory of Immunology (LIM 48), Hospital das Clínicas da Faculdade de Medicina of the University of São Paulo, São Paulo, Brazil; 3 Centro de Saúde Escola Barra Funda “Alexandre Vranjac”, Irmandade da Santa Casa de Misericórdia de São Paulo, São Paulo, Brazil; 4 Division of Infectious and Parasitic Diseases, Hospital das Clínicas da Faculdade de Medicina of the University of São Paulo, São Paulo, Brazil; 5 Hospital das Clínicas, Faculdade de Medicina Ribeirão Preto of the University of São Paulo, São Paulo, Brazil; 6 Centro de Vigilância Epidemiológica, Coordenadoria de Controle de Doenças da Secretaria da Saúde do Estado de São Paulo, São Paulo, Brazil; 7 Tropical Medicine Institute of São Paulo, University of São Paulo, São Paulo, Brazil; 8 Superintendência de Controle de Endemias, Secretaria da Saúde do Estado de São Paulo, São Paulo, Brazil; 9 Laboratory of Parasitology (LIM 46), Hospital das Clínicas da Faculdade de Medicina of the University of São Paulo, São Paulo, Brazil; 10 Control of Neglected Tropical Diseases, World Health Organization, Geneve, Switzerland; 11 Department of Social Medicine, School of Medical Sciences of Santa Casa de Misericórdia de São Paulo, São Paulo, Brazil; Liverpool School of Tropical Medicine, UNITED KINGDOM

## Abstract

**Background/Methods:**

In a pioneering cross-sectional study among Bolivian immigrants in the city of São Paulo, Brazil, the epidemiological profile, clinical manifestations and morbidity of Chagas disease were described. The feasibility of the management of Chagas disease at primary healthcare clinics using a biomedical and psychosocial interdisciplinary approach was also tested. Previously, a *Trypanosoma cruzi* (*T*. *cruzi*) infection rate of 4.4% among 633 immigrants was reported. The samples were screened using two commercial enzyme-linked immunoassay (ELISA) tests generated with epimastigote antigens, and those with discrepant or seropositive results were analyzed by confirmatory tests: indirect immunofluorescence (IFI), TESA-blot and a commercial recombinant ELISA. PCR and blood cultures were performed in seropositive patients.

**Results:**

The majority of the 28 seropositive patients were women, of whom 88.89% were of child-bearing age. The predominant clinical forms of Chagas disease were the indeterminate and atypical cardiac forms. Less than 50% received the recommended antiparasitic treatment of benznidazole. An interdisciplinary team was centered on primary healthcare physicians who applied guidelines for the management of patients. Infectologists, cardiologists, pediatricians and other specialists acted as reference professionals. Confirmatory serology and molecular biology tests, as well as echocardiography, Holter and other tests, were performed for the assessment of affected organs in secondary healthcare centers. The published high performance of two commercial ELISA tests was not confirmed.

**Conclusion:**

An interdisciplinary approach including antiparasitic treatment is feasible at the primary healthcare level for the management of Chagas disease in Bolivian immigrants. The itinerant feature of immigration was associated with a lack of adherence to antiparasitic treatment and was considered a main challenge for the clinical management of this population. This approach is recommended for management of the infected population in endemic and nonendemic areas, although different strategies are needed depending on the severity of the disease and the structure of the healthcare system.

## Author summary

Chagas disease affects approximately 6 million Latin American people. It is considered a neglected tropical disease since it mainly affects vulnerable, poverty-stricken people. Public health policies and investments in research on new treatment and control instruments have not been prioritized. In fact, disease urbanization occurred in Latin America in the 80s, and an estimated 15 million people moved from disease-endemic areas to nonendemic areas. Estimations have indicated that 2.9% of immigrants were infected by *T*. *cruzi* in 15 European countries, and more than 300,000 infected immigrants resided in the USA. In São Paulo, the estimated number of Bolivian immigrants exceeds 300,000. This study revealed the presence of mild clinical manifestations in predominantly young infected individuals, including reproductive-age women. For the first time, the feasibility of managing chronic Chagas disease at primary healthcare level centers with a biomedical and psychosocial interdisciplinary approach has been reported in the National Public Health System of Brazil. Additionally, the utilized commercial tests did not confirm the previously expected performance for diagnosis of infection. Finally, the itinerant characteristics of the Bolivian immigrant population was reported as a main factor underlying the lack of adherence to antiparasitic treatment and a main challenge for complete clinical management.

## Introduction

Chagas disease, which is caused by the protozoan flagellate parasite *T*. *cruzi* [1], affects approximately 6 million Latin American inhabitants of Mexico, Central and South America. It causes approximately 12,500 deaths annually, and 41,200 new cases are estimated each year [1–3].

Migration from disease-endemic areas to nonendemic areas within countries or between countries and continents, especially in the 20^th^ century, has led to the urbanization and globalization of Chagas disease. More than 15 million people from disease-endemic areas now live in nonendemic areas [4]. Approximately 2.9% (0.7–4.9%) [4, 5, 6] of immigrants were determined to be infected by *T*. *cruzi* in 15 European countries, excluding Spain [4]. Maternofetal transmission was estimated to occur in 0 to 3 of 4,000 newborns in nine European countries in 2009 [6]. The prevalence of infection in pregnant women varied from 4.7–17.7% and was higher in Bolivians [7]. In the USA, estimations have indicated that more than 300,000 individuals are infected [4]. In addition, approximately 3,600 infected individuals in Japan [8] and >3,000 in Australia [4] have been estimated.

As shown in Table 1, infection in immigrants varied from 0.62 to 10.3% [9–27] according to the age, to the center (blood banks, primary healthcare clinics, antenatal, maternity or specialized clinics), and the rate of infection was higher in Bolivian immigrants, ranging from 10.2 to 34.1% [9–25].

**Table 1:** Prevalence of *T*. *cruzi* infection in immigrants living in non endemic regions; screening and confirmatory tests.

Center	Patients—All countries or Bolivians	Number	Prevalence % All countries or Bolivians	Screening Serology	Confirmatory Serology	Newborn
Catalonia, Spain, 2008, Piron [9]	Blood banks Bolivian	1660 40	0.62 10.2	Agglutination+ELISApeptides		
Valencia, Spain, 2008, Parício Talayero [10]	Pregnant Bolivian Newborn	624 137	4.8 17.5 0	Immunoprecipitation (IP) Immunofluorescence (IF)	IF PCR IF	IF PCR
Barcelona, Spain, 2009. Soriano- Arandes [11]	Women CBA^a^ Bolivian Newborn	116 31 03	4.3 16.1 0	IC IC	rELISA^b^ ELISA whole Ag	
Valencia, Spain, 2009/2010, Orti- Lucas [12]	Pregnant Bolivian Newborn	400 77 37	9.3 26.0 2.7	IF IF	ELISA	Microhematocrit Hematocrit, PCR, IgM, IF
Barcelona, Spain, 2009, Muñoz [13] 2009a, Muñoz [14]	Pregnant Pregnan Newborn	1350 189 41	3.4 22.2 7.3	rELISA Crude Ag ELISA	ELISA 31.7 Lysate Ag	PCR, Microhematocrit
Houston, USA 2009, Di Pentima [15]	Pregnant Pregnant	2109 1658	0.4 Hispanic 0.1 non Hispanic	ELISA purified Ag	Hemagglutination	NR
Valencia, Spain, 2009, Lucas [16]	Pregnant Pregnant Newborn	388 77 27	9.7 26.0 2.7	IP	IF	
Geneva, Switzerland, 2009, Jackson [17]	Pregnant Bolivian	72 30	9.7 16.6	IF	Absent	
Geneva, Switzerland, 2010, Jackson [18]	General General	1012 485	12.8 26.2	2 commercialized ELISA	External control: 4 ELISA.,HA, IF	
Geneva, Switzerland, 2011, Jackson [19]	General Newborn Reactivation	253	253 04 01	IF or ELISA or IC		PCR,Microhematocrit Biopsy: amastigotes
Barcelona., Spain, 2011, Roca [20]	General Women ≥ 14y Bolivian	766 448 127	2.87 16.5	IC	rELISA, lysate ”in house”	
Spain, 2011 Perez-Ayala [21]	General Bolivian	1146 346	31.15	ELISA + IF		PCR 63%
Spain, 2012 Navarro et al. [22]	General Bolivian	217 203	21.7			
Valencia, Spain, 2012 Barona-Vilar [23]	Pregnant Bolivian Newborn	1975 628 216	11.4 34.1 3.7	2 ELISA or ELISA + IC or Agglutination	Second referred Test	Microhematocrit, IF IgM, PCRqualitative, PCR-quantitative
Spain, 2012 Ramos [24]	Pregnant Bolivian Paraguay	545 39 52	1.28 10.3 6.52	IP	IF PCR	
Elche, Spain, 2012, Ramos [25]	Pregnant Bolivian	566 73	9.59	IP, rELISA	cELISA^c^ or (cELISA+IF)	PCR
USA, 2009–11, Kopeluscznik [26]	Cardiopathy Bolivian	39 NR ^d^	13 NR	rELISA + IF	TESA blot	
São Paulo, Brazil, 2017, Luna et al, [27]	General Women CBA Children < 10y	633 279 11	4.4	2 ELISA epimastigotes Commercialized	rELISA TESA blot	

^a.^ CBA—child bearing age

^b.^ rELISA—recombinant ELISA

^c.^ cELISA—ELISA with crude antigens

^d.^ NR—not reported

To date, the majority of centers have reported a mild chronic Chagas disease (indeterminate form or mild cardiac forms) in approximately 2/3 of patients and less commonly the digestive or cardiodigestive form, as shown in Table 2 [10–14, 16–21, 28]. However, centers in Spain and the USA have also registered chronic Chagas disease patients with severe cardiopathy [21,26].

**Table 2:** Epidemiological characteristics, clinical forms of Chagas disease and antiparasitic treatment features in immigrants living in non endemic areas.

Center	Patients, Bolivians, Newborns	Number of Patients	Indeterminate Form %	Cardiac%	Digestive % (Cardiac or Digestive)	Treatment Drug mg/kg/d	Adverse Events	Treatment Interruption
Valencia, Spain, 2008, Paricio-Talayero [10]	Pregnant Bolivian Newborn	29 24 0	100	0	0	NR^a^	NR	NR
Barcelona, Spain, 2009, Soriano- Arandes [11]	Women Bolivian Newborn	5 0	NR	NR	NR	5 Benznidazole	Skin rash, Anorexia, Headache	NR
Barcelona, Spain, 2009 Munhoz [13]	Pregnant	46	100			NR	NR	NR
Barcelona, Spain, 2009a, Munhoz [14]	Pregnant Newborn^d^	41 03	Most NR	19	9	Benznidazole 7 mg/kg/d	NR	NR
Valencia, Spain, 2009, Orti-Lucas [16]	Pregnant Pregnant Newborn	40 20 01	NR NR	40.0^b^ 40.0^b^ Symptoms	31.6^b^ 31.6^b^ Symptoms	NR NR	NR NR	NR NR
Geneva, Jackson Switzerland, 2009, [17]	Pregnant Bolivian Newborn	07 05 02	Normal			Nifurtimox 10 mg/kgd-60d	NR	NR
Geneva,] Switzerland, 2010 Jackson [18]	General Bolivian	124 Majority	87.9	11.3	0.8	NR	NR	NR
Geneva, Switzerland, 2011, Jackson [19]	General Newborn Reactivation	253 04 01	71	19.8	1.6	129 (93—Nifurtimox)	56.2% Severe 7.4%	31.8%.
Barcelona, Spain 2011, Roca [20]	General Women	22 12	81	9.5	9.5 (Cardiac-Digestive)	NR	NR	NR
Madrid, Spain 2011,Perez-Ayala [21]	General Pregnant Newborn	252 11 0	196	17.11 Severe 5/43	3.5 (1.6)	195	52%	29.7%
Valencia, 2012, Barona-Vilar [23]	Pregnant Bolivian Newborn	226 214 08	NR 01 hepato splenomegaly	NR		NR Benznidazole 7mg/kg/d-60d	NR	NR
Japan, 2014, Imai [28]	Mother Maternofetal transmission	01 01			Megacolon	Benznidazole 5mg/kg/d 60d	Absent	No
Present work, Sao Paulo, Brazil, Shikanai-Yasuda, 2016	General Women Children ≤ 10y	28^d^ 17 ^d^ 03^d^	71.4 71.4	Non typical ECG^e^28.6	0	Benznidazole 9 patients 5mg/kg/d-60d	Dysgeusia 25%	No

^a.^ NR—not reported

^b.^ % positive serology in patients with cardiac or gastrointestinal symptoms

^c.^ Present work

^d.^ Childbearing age women

^e.^ ECG disturbances that are not common in chronic Chagas cardiopathy

In the 20^th^ century, the endemic area in Bolivia encompassed approximately 80% of the country, and the prevalence of infection was estimated to be 28.8% in 1988 [2,3]. This value varies from 4.9% to 51.0% among the general population according to the different districts [29], and from 17.3 to 70.5% among pregnant women, influencing the rates of maternofetal transmission [9]. Since 1990, intergovernmental initiatives of Latin American countries in South and Central America for the control and elimination of *Triatoma infestans* (*T*. *infestans*) and for the interruption of blood bank transmission have resulted in important changes in other countries of South and Central America. In Bolivia, the prevalence of infection in the general population [30] and in blood donors has decreased [31] similarly to the prevalence of infection in young children [32]. Vectorial transmission was interrupted in some districts (Departments of La Paz and Oruro).

A recent survey addressing congenital infection in Bolivia showed a prevalence of congenital infection ranging from 2–4%, in contrast to a previous value of 5% [33]. Estimates from PAHO-2006 [2] and WHO-2015 [1] indicated *T*. *cruzi* infection prevalences of 6.8% and 6.1%, respectively, in the general Bolivian population and 8.0% and 2.3%, respectively, in blood donors.

In parallel with Chagas disease nonendemic areas outside Latin America, the risk of *T*. *cruzi* transmission in the blood and its derivatives in nonendemic urban areas of Brazil is now very low: 0.21% of blood donors were infected [2], and vector-transmitted infection was detected in only 0.01% of children under 5 years of age from 11/2001 to 05/2008 [34]. Transmission by *T*. *infestans* has been under control in São Paulo State since the 70s [35,36,37] and in other endemic areas of this country since 2006 [2,38].

Taking into consideration the asymptomatic or oligosymptomatic condition of the majority of *T*. *cruzi*-infected patients and the lack of knowledge about Chagas disease, healthcare workers were not aware of Chagas disease, and the epidemiological background of the patient was not often investigated. Furthermore, serological tests were not performed for the early diagnosis of acute maternofetal transmission, infected blood donation, infected donors or recipients in organ transplantation or even Chagas disease reactivation in immunosuppressed hosts [5, 39].

The influx of Bolivian immigrants into Brazil started more than sixty years ago, initially through a cultural exchange program [40]. The Bolivian immigrant profile has changed since 1980 and more dramatically in recent years; it is characterized by people who work in sewing workshops (textile manufacturing) in the city of São Paulo under poor labor conditions (lack of contract security and labor rights, long working hours and low wages) [40, 41]. According to estimates published in the media, more than 300,000 documented and undocumented Bolivian immigrants live in the São Paulo Metropolitan Area, mostly in the city of São Paulo.

Only one prospective study on the prevalence of infection in Bolivian immigrants has been conducted in Brazil [27], and no studies were published on the access of the Bolivian population to local health systems for Chagas disease care. The arrival of a large number of Bolivian immigrants in São Paulo State introduced new scenarios regarding the epidemiology of Chagas disease and new questions related to disease control in urban centers where transmission interruption had already been consolidated.

The present study is part of a larger research project being conducted in São Paulo City that also describes the prevalence of infection in this population (27), their access to primary healthcare and to the reference centers for Chagas disease in the city of São Paulo and knowledge about Chagas disease of the target population.

We described a benign profile of chronic Chagas disease in Bolivian immigrants and noticed the itinerant immigration as a main characteristic of this population. We were unable to confirm the high performance of two commercial ELISA tests, one employed as a screening test and the other as a confirmatory test. Moreover, we reported the feasibility of this interdisciplinary approach centered on primary healthcare, the Brazilian Family Health program for management and antiparasitic treatment.

After reviewing the data on the prevalence of *T*. *cruzi* infection and morbidity in Bolivian immigrants in nonendemic areas, the aims of the present study were to describe the performance of screening and confirmatory serological tests and the clinical and epidemiological profiles of infected patients among Bolivian immigrants in the city of São Paulo. Additionally, we aimed to assess the feasibility of the management of Chagas disease at the primary healthcare level (Family Health Program of the Brazilian Ministry of Health) using a biomedical and psychosocial interdisciplinary approach and to test guidelines specifically prepared for the management of chronic Chagas disease at the primary healthcare level.

## Methods

Description of the epidemiological profile, clinical manifestations and Chagas disease morbidity of Bolivian immigrants in a primary healthcare center of the city of São Paulo. From 633 Bolivian immigrants previously screened for *T*. *cruzi* infection in a prevalence study for Chagas disease, those that were seropositive, seronegative or who had discrepant results were invited to participate in the present work in a Primary Healthcare Center of the city of São Paulo called “Centro de Saúde Escola Barra Funda Dr. Alexandre Vranjac—CSEBF” [27]. The CSEBF is a Primary Healthcare Center of the “Irmandade da Santa Casa de Misericórdia de São Paulo,” and it is part of the public national health system of Brazil (Sistema Único de Saúde—SUS) [42]. In São Paulo, the Family Health Program is implemented in partnership with the municipal health secretariat of São Paulo [43]. The CSEBF has been located in the Barra Funda district of the city of São Paulo since 1968 and is responsible for the primary healthcare of the families of this district. Bolivians live and work in sewing workshops in this area [42], and the center has a team that is responsible for approximately 1,000 Bolivian immigrants within an area comprising 18,000 inhabitants (IBGE, 2010) [44]. They receive visits from community health agents, physicians, nurses and other health workers. A Bolivian health agent is one component of this team.

### Ethics statement

The study was approved by the Ethics Committees of the “Hospital das Clínicas da Faculdade de Medicina” of the University of São Paulo and the School of Medical Sciences of “Santa Casa de Misericórdia” of São Paulo. All patients or the legal guardians of those under 18 years old signed an informed consent form to participate in the research.

### Interdisciplinary team for Chagas disease management

The community health agents and the clerical healthcare team worked actively to recruit seropositive patients from March 2014 to September 2015 and also to deliver the results of all patients serological tests for Chagas disease from February 2014 to October 2014.

The training of the health personnel team composed of physicians, nurses, laboratory workers and community agents was performed in five different phases: 1) initial meeting for training on ecoepidemiology, parasitology, pathology, diagnosis, treatment, follow-up, prevention and control of Chagas disease; 2) training of primary healthcare physicians on the management of Chagas disease by infectologists; 3) continued dialogue and discussion about the feasibility of the proposed approach as well as the reasons for the lack of adherence of patients to medical care and antiparasitic treatment; 4) continued supervision of the antiparasitic treatment and evolution of Chagas disease patients; 5) consolidation of the role of primary healthcare physicians to disseminate training for the management of Chagas disease in the same unit. They were able to prepare new physicians for the management of non-Bolivian patients who were infected with *T*. *cruzi*.

A pediatrician and a cardiologist who were accessible five days a week also provided support to the primary care team. Referral to a gastroenterologist and a specialized cardiology center to monitor digestive or cardiac function was possible during the project. Routine laboratory tests and tests for specific assessments of affected organs were also available during the follow-up period. Serological exams to confirm *T*. *cruzi* infection and tests to monitor parasitemia were performed in the Laboratory of Immunology and Laboratory of Parasitology of Hospital das Clínicas da Faculdade de Medicina, University of São Paulo, Brazil.

The interdisciplinary team was composed of primary healthcare physicians, infectologists, cardiologists, psychologists and epidemiologists from the Instituto of Tropical Medicine of São Paulo of the University of São Paulo and the Center for Epidemiologic Surveillance of São Paulo; researchers of health laws, public health and socio-anthropology; and biologists of zoonosis control from the “Superintendência de Controle de Endemias” in São Paulo State.

### Clinical forms and antiparasitic treatment—Primary healthcare center

Infected patients who were screened during the seroprevalence survey were referred to the Family Health Program physician. They were evaluated based on their clinical background and physical examination to search for signs of myocardiopathy, esophageal emptying disorders, megaesophagus or megacolon. Subsequently, they were subjected to a conventional electrocardiogram and thoracic and esophageal X ray. A double contrast barium enema was recommended in the case of constipation that lasted longer than one week. Patients were further classified as proposed by the Brazilian consensus [45] with: a) indeterminate form—without signs or symptoms and a normal ECG and thoracic and esophageal X rays; b) cardiac form—abnormalities found in Chagas disease patients [46]: right bundle-branch block, left anterior fascicular block, ST-T wave changes, electric inactive areas, abnormal Q waves or low QRS voltage, complex ventricular arrhythmias (polymorphic ventricular arrhythmias, couplet, non-sustained or sustained ventricular tachycardia), second degree or third degree (complete) atrioventricular block, junctional rhythm, atrioventricular dissociation and atrial fibrillation. An echocardiogram and 24-hour Holter were recommended and performed at a secondary healthcare level if clinical symptoms/signs or ECG abnormalities were detected; c) and digestive form if image data confirmed the presence of esophageal-emptying disturbances or megaesophagus or megacolon.

### Information provided to participants with negative serological results

Volunteers with negative serological results were invited to participate in a meeting with the interdisciplinary team to be informed about the results of the screening tests and about Chagas disease.

### Laboratory methods: Serology, parasitology and molecular biology assays

#### Serological screening tests

According to the international criteria established by the World Health Organization, sera that were reactive to *T*. *cruzi* antigens, as verified by two serological tests using different techniques and antigen targets, were considered positive (WHO, 2001) [47].

A case of *T*. *cruzi* infection was defined herein as an individual with a positive result in two different screening ELISA assays with epimastigote antigens, as confirmed by confirmatory tests. Sensitivity of serological tests was defined as reported [48].

Two commercial ELISA-based serological tests that were considered high-performance were selected as screening tests: ELISA test 1 (Chagas test ELISA III; Bioschile Ingenieria Genetica SA, Santiago, Chile) and ELISA test 2 –(ELISA *cruzi*; BioMerieux Diagnostics SA, Rio de Janeiro, Brazil).

#### Serological confirmatory tests

Three confirmatory tests were employed in the case of seropositive results for both ELISA screening tests or discrepant results (positive and negative or doubtful results for any test) for ELISA 1 or ELISA 2:

1. Immunoblot with trypomastigote antigens (TESA blot)—This test was performed as previously described [27, 48, 49].
2. ELISA 3—Recombinant ELISA with trypo and epimastigote antigens (ELISA Chagatest^®^ Wiener Lab, Rosario, Argentina) conducted according to the manufacturer’s instructions and as previously described [27, 48].
3. Indirect immunofluorescence test (IFI)—Performed with epimastigote parasites that were pre-adsorbed onto immunofluorescence glass slides [48]. The results were observed under a fluorescence microscope at 400x magnification and a cutoff of 1/40 was considered. Positive and negative controls were assessed.

#### Negative results

Samples wth negative results by both screening tests (ELISA 1 and ELISA 2) and those with discrepant results by screening tests that were not confirmed by confirmatory tests were considered negative as defined by the WHO (2010) [48].

### Parasitological and molecular tests

The blood culture assay was performed as previously described by Luz et al., 1994 [50]. Six culture tubes were examined after 30, 60 and 90 days of culture. The results were expressed as positive if at least one tube was positive and negative if all were negative.

#### Molecular tests

Qualitative PCR was performed using the kDNA sequence as described by Avila et al. [51] with modifications. Briefly, DNA was extracted using the QIAampTM DNA Mini Kit (Qiagen, Hilden, Germany) from whole blood and/or serum collected from seropositive patients and those with inconclusive serological results whose whole blood was not accessible. The S35 and S36 primer pair was used to amplify a 330-bp parasite minicircle sequence (GibcoTM Life Technologies, CA, USA). The reactions contained Taq polymerase, 0.2 mM of each primer, 2.9 mM MgCl_2_ and 50–150 ng of DNA. The following controls were used: negative controls for the master mix preparation and DNA addition; a positive control consisting of 2.10–15 g DNA from the Y strain of *T*. *cruzi* and inhibition controls of DNA amplification, as verified by amplification of duplicate patient samples containing parasite DNA. The analytical sensitivity of this assay was 0.2 fg of *T*. *cruzi*, which corresponds to 0.01 parasite/assay in an agarose gel. Quantitative PCR was performed as previously described by Freitas et al. [52] with the microsatellite sequence TCZ3/TCZ4 as internal primers for TCZ1 and TCZ2 (GibcoTM Life Technologies, CA, USA) and using 10 μL SYBR Advantage qRT-P (Clontech, CA, USA), according to the manufacturer’s instructions. The mixture (20 μL) contains *Taq* polymerase, 0.2 mM of each primer, 2.9 mM MgCl_2_ and 50–150 ng of DNA.

### Statistical analyses

To perform the statistical analysis, version 20.2 of the SPSS^®^ software was used. Descriptive statistics and 95% confidence intervals for proportions were calculated, whenever appropriate.

### Outcomes

The main outcomes included the following: a) sensitivity of serological screening tests and comparison among perfomances of serological confirmatory tests in seropositive or discrepant results; b) distribution of infected patients according to clinical forms; c) distribution of infected patients according to the departments where they lived in Bolivia; d) proportion of treated patients and reason for non-adherence; e) frequency of adverse events related to the treatment; f) feasibility of this interdisciplinary approach at the primary healthcare level in the National Public Health System of Brazil for the management of Chagas disease; and g) usefulness of guidelines specifically prepared for this approach.

## Results

### Performance of screening and confirmatory serological tests for *T*. *cruzi* infection: Results of screening tests and confirmatory serological tests

To analyze the performance of different serologies, data from twenty eight seropositive samples analyzed by two screenings and three confirmatory tests are presented in Table 3. The recombinant ELISA test using trypo and epimastigote antigens with a confirmatory value revealed a lower sensitivity (96.4%) than the other confirmatory tests: the TESA-blot and IF.

**Table 3:** Results of screening and confirmatory tests for *T*. *cruzi* antigens in 28 samples with seropositive results and in 18 samples with discrepant results by screening tests.

Serology	Screening tests: Positive results			Screening tests: Discrepant results		
Screening	N^1^	% +/N	%Neg^2^/N	N	% +/N	% Neg/N
ELISA 1	28	100	0	18	0	100
ELISA 2	28	100	0	18	100^3^	0
**Confirmatory**			0			
Immunoblot	28	100		18	0	100
mmunofluorescence	28	100	0	18	5.6	94.4
ELISA 3	28	96.4^4^	0	18	100	100

^1-^N = Number of samples (one per patient)

^2-^Neg = Negative

^3^ ELISA 2–18 positive results in 605 seronegative samples by ELISA 1, none confirmed by at least two confirmatory tests

^4^ Sensitivity = 96.4% (95%CI: 89.54–100.0)

### Discordant serological results

Sera from eighteen patients presented discordant results by the screening tests ELISA 1 and 2, and seven of them were from children <2 years old. All the serological tests showed a similar frequency of negative results, excluding ELISA 2, which presented 18 seropositive results and IF, which revealed one seropositive result without confirmation by at least two confirmatory tests (Table 3). Thus, 605 samples were considered negative (587 samples were negative by ELISA 1 and 2, and 18 samples had discrepant results but negative results in at least two confirmatory tests).

Qualitative PCR did not reveal *T*. *cruzi* kDNA over the detection limit in any of the 18 serum samples. A second sample available from seven volunteers with discrepant results revealed concordant serum-negative results with the two screening ELISA 1 and 2 tests and the three confirmatory tests and in whole blood by PCR.

### Parasitemia detected by blood culture and PCR

Blood cultures were positive in 23.04% (95% CI: 0.2–46.0) of patients with seropositive results; qualitative/quantitative PCR was positive in 30.8% (95% CI: 5.7–55.9), and either blood culture or PCR was positive in 46.2% (95% CI: 19.1–73.3) of them.

### Patient management

#### Information given to patients with negative serological results

Six hundred and five volunteers (or their representatives) with negative results received the results of their serological tests in educational meetings in which they were informed about Chagas disease by a multidisciplinary team consisting of infectologists, nurses and epidemiologists. They also received a folder containing basic data about epidemiology, diagnosis, control and prevention of this disease as well as a recommendation to seek primary healthcare in the case of symptoms or signs. This folder was also given to all study participants with positive or inconclusive results.

#### Adherence to the proposed schedule of medical consultations and antiparasitic treatment of patients with positive serological results

Twenty eight volunteers diagnosed with Chagas disease were invited to attend a medical consultation at the primary healthcare center. Clinical forms were characterized based on a physical examination and complementary exams to assess target organs of *T*. *cruzi* as described in the Methods. When indicated, according to Brazilian Guidelines of Chagas disease [45], antiparasitic medicines and follow-up treatment were provided at this level. Eighteen patients (64.29%) attended the first medical consultation. For those who did not, a second or even a third consultation with a physician (including a Bolivian infectologist) was scheduled during visits to the patient’s home or workplace (10 patients). The same schedule was repeated for patients who were absent at later follow-ups (6 patients).

In addition to communication of the laboratory results, the main objective of the consultations was to explain to patients the disease evolution and the opportunity to manage Chagas disease before it develops into severe tissue involvement.

A search in the database of the São Paulo health municipal system was also performed to identify 15 patients who may have changed their addresses within the city. A phone call to Bolivia (by a Bolivian member of the team) was made to search for patients who may have returned to their country of origin. Finally, a letter was sent to the Bolivian Consulate in São Paulo, aiming to notify patients about the results of the serological tests. We were not successful in these endeavors; therefore, the information obtained for these patients was provided by their friends or neighbors.

#### Guidelines for Chagas disease management at the basic healthcare level and mobile app

As an instrument to train primary healthcare physicians about the management of patients with chronic Chagas disease, a guideline was prepared by our team and recommended to healthcare physicians [46].

This guideline was tested by primary healthcare physicians of CSEBF who managed the patients and was approved by this team for the training of other physicians in this and other Family Health Units. Furthermore, it has been applied to the management of non-Bolivian patients with Chagas disease by other clinicians of CSEBF. It was accessible through a print version in Portuguese and through two “on-line” versions in Portuguese and Spanish languages [46].

#### Mobile app

This guide was designed as a tool to help clinicians diagnose and treat Chagas disease at the primary healthcare level.

### Characteristics of the studied seropositive Bolivian immigrants: Epidemiological data

Women of child-bearing (10–49 years of age) age represented 88.89% of the total number of seropositive women. The main Bolivian Departments where the patients were born are shown in Table 4. From seropositive patients, 21.4% had mothers who were seropositive to *T*. *cruzi* antigens, but most of them lived in endemic areas. Triplets of 4.5 years old from a seropositive mother lived in the Department of Santa Cruz for 3.5 years. Thus, maternofetal transmission could be possible, but vector-transmitted infection could not be excluded.

**Table 4:** Departments of birth in Bolivia of seropositive Bolivian immigrants.

Number of Patients and Departments in Bolivia			
Departments	Rural	Urban	Total
**Santa Cruz de La Sierra**	4	7	11
**La Paz**	0	7	7
**Cochabamba**	1	3	4
**Chuquisaca**	2	1	3
**Potosia dupl**	2	0	2
**Oruro**	1	0	1
**Total**	10	18	28

### Clinical forms of Chagas disease and antiparasitic treatment

Among the 18 patients who attended the first medical consultation at the primary healthcare level, 72.2% were women, 77.7% had an electrocardiogram, 66.7% had a thoracic X-ray and 72.2% had an esophageal X-ray with contrast. Only one patient was referred to a secondary healthcare level to perform an echocardiogram. The data showed that 71.42% had an indeterminate form, and 28.57% had electrocardiographic abnormalities that are not commonly reported in Chagas disease (disturbances in ventricular repolarization and sinus arrhythmia). No involvement of the digestive system was observed.

Only 44.4% of the patients who attended the first medical consultation received antiparasitic treatment with benznidazole 5 mg/kg/day up to 300 mg/day for 60 days (N = 8). Women of child-bearing age represented 87.2% of treated patients. Follow-up period after the treatment varied from 2–15 months. The 9^th^ patient moved to another area of the city and did not return after 15 days of treatment. Concerning adverse events, dysgeusia was referred by 37.5% and gastrointestinal disturbances by 12.5%. One patient whose therapy was interrupted on day 56 due to ageusia fully recovered her healthy status 14 days later. Serology remained positive in two treated patients one year after the treatment, and one of two patients who were PCR-positive in the pretreatment period became negative.

Four of the five male patients and five of the 13 women did not receive benznidazole. The main reasons for medical appointment non-attendance or antiparasitic treatment refusal are shown in Table 5.

**Table 5:** Reasons for antiparasitic treatment refusal or for medical appointment non attendance.

Reasons	% of patients	(N = 19)
**Returned to Bolivia**		36.84
**Refused to be treated**		21.05
** Fear of job loss**	5.26	
** Preference to be treated in Bolivia**	5.26	
** Unknown reasons**	10.52	
**Moved to another area of the city**		5.26
**Generalized dermopathy**		5.26
**Abandonment on the 3^rd^ day of treatment**		5.26
**Patient not found, no available information**		26.32
**Total**		99.99

As shown in Table 5, itinerant immigration from Bolivia-São Paulo-Bolivia was identified as the main reason for the lack of adherence to medical follow-up and antiparasitic treatment.

## Discussion

A large number of Bolivian immigrants arrive in the city of São Paulo, mainly because of the availability of temporary work, causing changes in the epidemiological profile of Chagas disease in a nonendemic area within an endemic country. A prevalence of *T*. *cruzi* infection of 4.4% has been found in this Bolivian population [27], thus introducing new public health challenges regarding the control of the disease through blood derivatives, organ transplants or maternofetal transmission. The risk of chronic disease reactivation in immunosuppressed patients must also be considered in this new context.

Considering the young age of the Bolivian immigrants and the high proportion of child-bearing age women, serological tests to diagnose this infection in women of child-bearing age and/or who are pregnant should be implemented.

In our study, both screening tests with epimastigote antigens, selected by their high performance for the diagnosis of Chagas disease, have shown high sensitivity (100%) in comparison to other screening tests employed in Europe [53,54]. However, ELISA 2 provided false-positive results at a rate of approximately 3%. The TESA-blot had the best performance in terms of sensitivity (100%) and specificity. It is also able to distinguish between antibodies against *T*. *cruzi* and against *Leishmania* sp. [48]. Nevertheless, this test is expensive and might not be as accessible as other commercial tests (TESA-blot production was recently interrupted by Bio-Mérieux). IF had the same sensitivity but provided one false-positive result in relationship to the other tests. ELISA 3 was also employed as a confirmatory test in the search for an accessible commercial test with high sensitivity and specificity due to the inclusion of both trypomastigote and epimastigote recombinant antigens. However, this ELISA test showed a low sensitivity (96.4%) and is not recommended as a screening test. In our study, the best screening test was ELISA 1, and the performance of ELISA 2 and 3 was lower than expected [48].

The rate of PCR positivity (30.8%) observed herein was lower than that previously described (41.6%) in a similar small sample of Bolivian infected patients [55]. This finding could be related to the presence of the TcV molecular type, for which the RT PCR primers used in this work were less sensitive. This TcV type was commonly reported in the districts of Bolivia in which the patients lived [56,57]. Moreover, PCR inhibition was excluded through the use of positive controls.

Regarding the child-bearing age women (10–49 years) analyzed in the present study, the described prevalence of 6.1% [27] was higher than that described by Munhoz et al. [13] but lower than those registered in Europe (Tables 1 and 2) [10,11,12,14,17,23,24]. Moreover, in recent and previous publications, a higher prevalence was registered for general Bolivian immigrants in European countries [5,12, Table 1] compared with São Paulo [27]. The patients evaluated in the present study migrated recently from Bolivia (0–5 years) [27], and most of them were young adults that lived in the urban area of La Paz. Thus, they might be less exposed to infection because the prevalence of *T*. *cruzi* infection in that area was lower than in other Bolivian Departments. Another explanation for the lower prevalence found in our study could be related to the screening centers. In fact, our work was prospectively performed in a primary healthcare center for the general population, which was perhaps more representative of the general immigrant population than antenatal clinics, maternity hospitals or Tropical Medicine centers.

As reflected in Table 2, both in our work and in the majority of reports in the literature, most of the patients presented a benign form of the disease in the chronic phase (indeterminate form). Mild electrocardiographic atypical disturbances were reported in 28.57% of cases, none of which had typical or severe cardiopathy. Cardiac involvement was reported in 9.0–19.8% of some centers outside the endemic area [14,18,19,21], sometimes with severe cardiopathy [21], as shown in Table 2. However, the electrocardiographic disturbances were not commonly described for comparison to those found in the present analysis.

Regarding the management of chronic Chagas disease, access to diagnosis and treatment facilities for Chagas disease/infection is proposed to occur at different healthcare levels.

In our work, diagnosis using screening tests, detection of affected organs, definition of chronic clinical forms and antiparasitic treatment for mild cases took place at the first primary healthcare center. Support of some specialties (infectologists, cardiologists and pediatricians) as well as the entire interdisciplinary team was accessible at this level, as previously recommended [58].

For the management of chronic cardiac and non-cardiac forms, primary healthcare physicians were trained regarding the correct interpretation of electrocardiographic abnormalities attributed to Chagas disease or to other cardiopathies. Mild chronic Chagas disease in young Bolivian immigrants, predominantly child-bearing age women, was easily managed in primary healthcare centers by a multidisciplinary team. This experience focusing on primary healthcare physicians of the Brazilian Family Health Program had been highly successful. Similar experiences in other contexts have been reported by “Médecins Sans Frontières (MSF)/Doctors Without Borders” in Latin American countries [59].

At the secondary healthcare level, we performed parasitological, molecular and confirmatory serological tests for positive and discrepant samples and more complex complementary exams for functional organ evaluations. Reference centers for severe adverse effects of antiparasitic treatment and specialized support for severe forms of the disease were also available. However, no patients were referred due to the absence of these effects or to such severe clinical manifestations.

At the third healthcare level, further interventions for more severe cardiopathy or complicated digestive forms (pacemakers, transplants or untreatable cardiac heart failure; cardiac arrhythmia; or surgery for megaesophagus or megacolon) were planned but were not registered.

In summary, we validated the feasibility of the management of chronic Chagas disease by primary healthcare physicians of the Family Health Program of the National Health System. We suggest that this Program could be responsible for the management of non-severe chronic Chagas disease and its antiparasitic treatment. All necessary support for more severe cases could be provided by specialized centers and reference laboratories.

One important limitation affecting our study was the lack of patient adherence to the treatment, which had the effect of further reducing the sample size. The main associated factor was the itinerant immigration via the Bolivia-São Paulo State-Bolivia route, which remains one of the greatest challenges for future approaches. We hope that a direct link established with the “Programa Nacional de Chagas, Unidad de Epidemiologia, Ministerio del Salud, Bolivia” could implement mutual cooperative actions for the management and antiparasitic treatment of these immigrants. Moreover, as fear of job loss is one cause of lack of adherence, we suggest a more flexible schedule for medical appointments. A similar effect has been observed due to the absence of legal documents, which has been the focus of our project on access to healthcare. Our recommendation was to capacitate primary health services to orient the immigrant regarding the documentary regularization for their permanence in the country.

The sustainability of the project was promoted through the training of the health personnel to act as multipliers. Guidelines are now accessible “on- and off-line” [46] and shown to be useful to healthcare physicians for Chagas disease management at the primary care level. The mobile app is also accessible to help physicians through continuous education in epidemiological and clinical aspects, diagnosis and antiparasitic treatment of Chagas disease.

Considering the emergence of new epidemiological scenarios introduced through the movement of the immigrant-infected population and the results of the present study in terms of the management of chronic Chagas disease, new challenges in the organization of Brazilian health services for primary and specialized healthcare are: a) to train primary healthcare physicians in the management of chronic Chagas disease for approximately one million infected Brazilian people as well as for the infected immigrant population; b) to clearly indicate specialized reference centers for Chagas disease care; and c) to achieve the approval of specific clinical and therapeutic protocols by the Health Ministry to implement diagnostic and antiparasitic treatment strategies throughout the country.

Finally, the training of health professionals and undergraduate students through the implementation of education in the nuclear curriculum must be continuously stimulated.

## Supporting information

S1 Annex Demographic and epidemiological data basis. (XLSX)

S2 Annex Clinical data basis. (XLSX)

S1 Checklist Strobe checklist. (DOC)
