# Supplementary material for: Interdisciplinary approach at the primary healthcare level for Bolivian immigrants with Chagas disease in the city of São Paulo
Source: PLoS Negl Trop Dis. 2017 Mar 23;11(3):e0005466. doi: 10.1371/journal.pntd.0005466 (PMC5380346; doi:10.1371/journal.pntd.0005466)
Supplement: S1 Checklist — (DOC) [file pntd.0005466.s003.doc]

STROBE Statement—Interdisciplinary approach at the primary healthcare level for Bolivian immigrants with Chagas disease in the city of São Paulo

|  | Item No | Recommendation | Lines |
| --- | --- | --- | --- |
| **Title and abstract** | 1 | (*a*) Indicate the study’s design with a commonly used term in the title or the abstract | 50 |
|  |  | (*b*) Provide in the abstract an informative and balanced summary of what was done and what was found | 50-54  55-73 |
| Introduction | | |  |
| Background/rationale | 2 | Explain the scientific background and rationale for the investigation being reported | 54-55  153-157  171-183 |
| Objectives | 3 | State specific objectives, including any prespecified hypotheses | 195-201 |
| Methods | | |  |
| Study design | 4 | Present key elements of study design early in the paper | 205-209  55-58 |
| Setting | 5 | Describe the setting, locations, and relevant dates, including periods of recruitment, exposure, follow-up, and data collection | 213-217  226-228  447-448 |
| Participants | 6 | (*a*) Give the eligibility criteria, and the sources and methods of selection of participants | 205-209  254-270  281-305 |
| Variables | 7 | Clearly define all outcomes, exposures, predictors, potential confounders, and effect modifiers. | 332-340 |
| Data sources/ measurement | 8* | For each variable of interest, give sources of data and details of methods of assessment (measurement). Describe comparability of assessment methods if there is more than one group | 255-270  278-327  378-384 |
| Bias | 9 | Describe any efforts to address potential sources of bias | 300-301  317-320  378-386  391-396  402-406 |
| Study size | 10 | Explain how the study size was arrived at | 205-209  378-385  426-427 |
| Quantitative variables | 11 | Explain how quantitative variables were handled in the analyses. If applicable, describe which groupings were chosen and why | 329-330  364-366  385  426-429 |
| Statistical methods | 12 | (*a*) Describe all statistical methods, including those used to control for confounding | 329-330 |
|  |  | (*b*) Describe any methods used to examine subgroups and interactions | Not applicable |
|  |  | (*c*) Explain how missing data were addressed | 394-398  402-406 |
|  |  | (*d*) If applicable, describe analytical methods taking account of sampling strategy | Not applicable |
|  |  | (*e*) Describe any sensitivity analyses | 364-366  385 |
| Results | | |  |
| Participants | 13* | (a) Report numbers of individuals at each stage of study—eg numbers potentially eligible, examined for eligibility, confirmed eligible, included in the study, completing follow-up, and analysed | 390-391  395-396  438-446 |
|  |  | (b) Give reasons for non-participation at each stage | 458-460 |
|  |  | (c) Consider use of a flow diagram | Not applicable |
| Descriptive data | 14* | (a) Give characteristics of study participants (eg demographic, clinical, social) and information on exposures and potential confounders | 425- 444  445-449 |
|  |  | (b) Indicate number of participants with missing data for each variable of interest | 438-440  445-448  454-460 |
| Outcome data | 15* | Report numbers of outcome events or summary measures | 378-385  441-450  458-460  539-540 |
| Main results | 16 | (*a*) Give unadjusted estimates and, if applicable, confounder-adjusted estimates and their precision (eg, 95% confidence interval). Make clear which confounders were adjusted for and why they were included / | 329-330  364-366  385 |
|  |  | (*b*) Report category boundaries when continuous variables were categorized | Not applicable |
|  |  | (*c*) If relevant, consider translating estimates of relative risk into absolute risk for a meaningful time period | Not applicable |
| Other analyses | 17 | Report other analyses done—eg analyses of subgroups and interactions, and sensitivity analyses | Not applicable |
| Discussion | | |  |
| Key results | 18 | Summarise key results with reference to study objectives | 475-488  507-510  539-543  555-559 |
| Limitations | 19 | Discuss limitations of the study, taking into account sources of potential bias or imprecision. Discuss both direction and magnitude  of any potential bias | 544-554 |
| Interpretation | 20 | Give a cautious overall interpretation of results considering objectives, limitations, multiplicity of analyses, results from similar studies, and other relevant evidence | 523-528  539-544  560--568 |
| Generalisability | 21 | Discuss the generalisability (external validity) of the study results | 414-417  544-545 |
| Other information | | |  |
| Funding | 22 | Give the source of funding and the role of the funders for the present study and, if applicable, for the original study on which the present article is based | Plos Neg  Trop Dis form |

*Give information separately for exposed and unexposed groups.

**Note:** An Explanation and Elaboration article discusses each checklist item and gives methodological background and published examples of transparent reporting. The STROBE checklist is best used in conjunction with this article (freely available on the Web sites of PLoS Medicine at http://www.plosmedicine.org/, Annals of Internal Medicine at http://www.annals.org/, and Epidemiology at http://www.epidem.com/). Information on the STROBE Initiative is available at www.strobe-statement.org.
